# 3-(3-Bromo­benz­yl)isoquinolin-1(2*H*)-one

**DOI:** 10.1107/S1600536809051137

**Published:** 2009-12-04

**Authors:** Farukh Iftakhar Ali, Tariq Mahmood Babar, Nasim Hasan Rama, Peter G. Jones

**Affiliations:** a Department of Chemistry, Quaid-i-Azam University, Islamabad 45320, Pakistan; b Institut für Anorganische und Analytische Chemie, Technische Universität Braunschweig, Postfach 3329, 38023 Braunschweig, Germany

## Abstract

In the title compound, C_16_H_12_BrNO, the ring systems subtend an inter­planar dihedral angle of 75.95 (3)°. In the crystal packing, mol­ecules are linked to form centrosymmetric pairs by pairs of classical N—H⋯O hydrogen bonds.

## Related literature

For the biological and pharmaceutical properties of isoquinolin-1(2*H*)-one derivatives, see: Chern & Li (2004 ▶); Coelho *et al.* (2003 ▶); Jayaraman *et al.* (2000 ▶); Thompson & Kallmerten (1990 ▶); Ukita *et al.* (2001 ▶). For the structure of a related isochromene derivative, see: Ali *et al.* (2009 ▶).
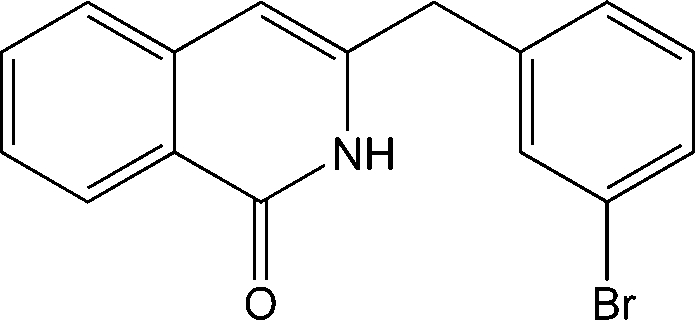


## Experimental

### 

#### Crystal data

C_16_H_12_BrNO
*M* *_r_* = 314.18
Triclinic, 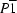

*a* = 4.5858 (4) Å
*b* = 9.4976 (7) Å
*c* = 14.8296 (11) Å
α = 88.698 (6)°
β = 83.829 (6)°
γ = 86.529 (6)°
*V* = 640.88 (9) Å^3^
*Z* = 2
Cu *K*α radiation
μ = 4.28 mm^−1^
*T* = 100 K
0.16 × 0.07 × 0.07 mm

#### Data collection

Oxford Diffraction Nova A diffractometer
Absorption correction: multi-scan (*CrysAlis RED*; Oxford Diffraction, 2009 ▶) *T*_min_ = 0.817, *T*_max_ = 1.000
9267 measured reflections
2642 independent reflections
2586 reflections with *I* > 2σ(*I*)
*R*_int_ = 0.021

#### Refinement

*R*[*F* ^2^ > 2σ(*F* ^2^)] = 0.026
*wR*(*F* ^2^) = 0.068
*S* = 0.90
2642 reflections
176 parameters
H atoms treated by a mixture of independent and constrained refinement
Δρ_max_ = 0.54 e Å^−3^
Δρ_min_ = −0.65 e Å^−3^

### 

Data collection: *CrysAlis CCD* (Oxford Diffraction, 2009 ▶); cell refinement: *CrysAlis RED* (Oxford Diffraction, 2009 ▶); data reduction: *CrysAlis RED*; program(s) used to solve structure: *SHELXS97* (Sheldrick, 2008 ▶); program(s) used to refine structure: *SHELXL97* (Sheldrick, 2008 ▶); molecular graphics: *XP* (Siemens, 1994 ▶); software used to prepare material for publication: *SHELXL97*.

## Supplementary Material

Crystal structure: contains datablocks global, I. DOI: 10.1107/S1600536809051137/rz2396sup1.cif

Structure factors: contains datablocks I. DOI: 10.1107/S1600536809051137/rz2396Isup2.hkl

Additional supplementary materials:  crystallographic information; 3D view; checkCIF report

## Figures and Tables

**Table 1:** Hydrogen-bond geometry (Å, °)

*D*—H⋯*A*	*D*—H	H⋯*A*	*D*⋯*A*	*D*—H⋯*A*
N—H01⋯O^i^	0.85 (3)	1.96 (3)	2.8036 (19)	176 (2)

Symmetry code: (i) 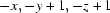.

**Fig. 1.**
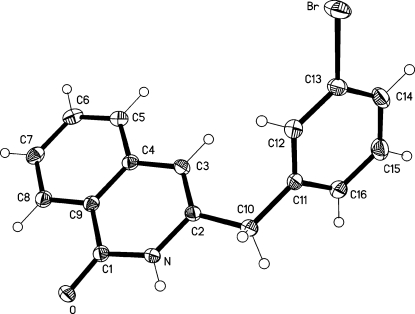
Molecular structure of the title compound showing the atom labelling scheme and displacement ellipsoids at the 50% probability level.

**Fig. 2.**
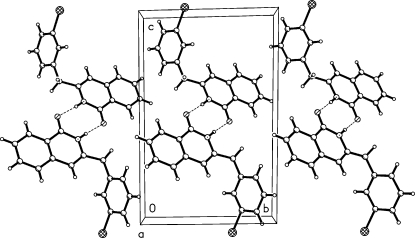
Packing diagram viewed parallel to the x axis. Hydrogen bonds are indicated by dashed lines.

**Fig. 3.**
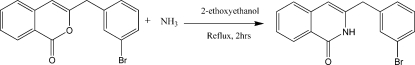
The formation of the title compound.

## supplementary crystallographic information

### Comment

Isoquinolin-1(2*H*)-one derivatives are an important class of heterocyclic compounds with substantial biological activities (Jayaraman *et al.*, 2000) that can be found in naturally occurring products of medicinal interest (Ukita *et al.*, 2001) and synthetic pharmaceuticals such as thalifoline (Chern & Li, 2004), pancratistain and lycoricidine (Thompson & Kallmerten, 1990). In addition, isoquinolin-1(2*H*)-ones are versatile building blocks for the total synthesis of natural isocarbostyril alkaloids (Coelho *et al.*, 2003). Bearing in mind the pharmaceutical importance of this class of compounds, the title compound, an isoquinolinone derivative containing a 3-bromobenzyl substituent, has been synthesized and its crystal structure is reported here. We have also determined the structure of the analogous isochromene derivative with an oxygen atom replacing the NH group (Ali *et al.*, 2009).

The molecule of the title compound is shown in Fig. 1. Bond lengths and angles may be regarded as normal. The atom sequence N—C2—C10—C11 displays a *trans* geometry, with a torsion angle of -178.69 (14). The two planar ring systems (including all non-hydrogen substituents) are both planar to within r.m.s. deviations of 0.01 Å and subtend an interplanar angle of 75.95 (3)°. As in the analogous isochromene derivative (Ali *et al.*, 2009), several bond angles depart substantially from ideal values, *e.g.* C1—N—C2 125.36 (14), N—C2—C10 113.15 (14), C2—C2—C10 126.87 (15), C2—C10—C11 114.88 (14)°.

The packing diagram (Fig. 2) shows the molecules to be linked by classical hydrogen bonds N—H···OC across inversion centres.

### Experimental

3-(3'-Bromobenzyl)isocoumarin (1 g, 0.0032 mol) in 2-ethoxyethanol was saturated with ammonia gas for 2 h, forming a pale yellow solution that was refluxed for 2 h. The solvent was evaporated under reduced pressure, yielding a fluffy solid. This that was purified by column chromatography using 50% ethyl acetate/petroleum ether as an eluent to afford the title compound (yield 61%; m.p. 228–230 °C). Crystals suitable for X-ray analysis were obtained by slow evaporation of an ethyl acetate solution.

### Refinement

The H atom bount to the nitrogen atom was refined freely. Other H atoms were placed in calculated positions and refined using a riding model with C—H = 0.95–0.99 Å and with *U*_iso_(H) = 1.2 *U*_eq_(C).

### Crystal data

**Table:**

C_16_H_12_BrNO	*Z* = 2
*M**_r_* = 314.18	*F*(000) = 316
Triclinic, *P*1	*D*_x_ = 1.628 Mg m^−^^3^
Hall symbol: -P 1	Cu *K*α radiation, λ = 1.54184 Å
*a* = 4.5858 (4) Å	Cell parameters from 9315 reflections
*b* = 9.4976 (7) Å	θ = 3.0–75.6°
*c* = 14.8296 (11) Å	µ = 4.28 mm^−^^1^
α = 88.698 (6)°	*T* = 100 K
β = 83.829 (6)°	Prism, colourless
γ = 86.529 (6)°	0.16 × 0.07 × 0.07 mm
*V* = 640.88 (9) Å^3^	

### Data collection

**Table:**

Oxford Diffraction Nova A diffractometer	2642 independent reflections
Radiation source: Nova (Cu) X-ray Source	2586 reflections with *I* > 2σ(*I*)
mirror	*R*_int_ = 0.021
Detector resolution: 10.3543 pixels mm^-1^	θ_max_ = 75.8°, θ_min_ = 3.0°
ω scan	*h* = −5→5
Absorption correction: multi-scan (*CrysAlis RED*; Oxford Diffraction, 2009)	*k* = −11→11
*T*_min_ = 0.817, *T*_max_ = 1.000	*l* = −18→18
9267 measured reflections	

### Refinement

**Table:**

Refinement on *F*^2^	Primary atom site location: structure-invariant direct methods
Least-squares matrix: full	Secondary atom site location: difference Fourier map
*R*[*F*^2^ > 2σ(*F*^2^)] = 0.026	Hydrogen site location: inferred from neighbouring sites
*wR*(*F*^2^) = 0.068	H atoms treated by a mixture of independent and constrained refinement
*S* = 0.90	*w* = 1/[σ^2^(*F*_o_^2^) + (0.0429*P*)^2^ + 0.8338*P*] where *P* = (*F*_o_^2^ + 2*F*_c_^2^)/3
2642 reflections	(Δ/σ)_max_ = 0.016
176 parameters	Δρ_max_ = 0.54 e Å^−^^3^
0 restraints	Δρ_min_ = −0.65 e Å^−^^3^

### Special details

**Table:**

Geometry. All e.s.d.'s (except the e.s.d. in the dihedral angle between two l.s. planes) are estimated using the full covariance matrix. The cell e.s.d.'s are taken into account individually in the estimation of e.s.d.'s in distances, angles and torsion angles; correlations between e.s.d.'s in cell parameters are only used when they are defined by crystal symmetry. An approximate (isotropic) treatment of cell e.s.d.'s is used for estimating e.s.d.'s involving l.s. planes.Least-squares planes (*x*,*y*,*z* in crystal coordinates) and deviations from them (* indicates atom used to define plane)- 3.3521 (0.0017) *x* + 6.0480 (0.0040) *y* - 1.1834 (0.0042) *z* = 2.8164 (0.0038)* 0.0154 (0.0011) C10 * -0.0101 (0.0014) C11 * -0.0109 (0.0014) C12 * -0.0098 (0.0015) C13 * 0.0036 (0.0015) C14 * 0.0074 (0.0013) C15 * -0.0053 (0.0014) C16 * 0.0097 (0.0008) BrRms deviation of fitted atoms = 0.00963.3793 (0.0010) *x* + 4.6226 (0.0023) *y* + 8.8098 (0.0043) *z* = 6.7949 (0.0012)Angle to previous plane (with approximate e.s.d.) = 75.95 (0.03)* 0.0161 (0.0013) C10 * -0.0154 (0.0013) N * 0.0007 (0.0011) O * -0.0018 (0.0014) C1 * -0.0080 (0.0015) C2 * -0.0054 (0.0015) C3 * 0.0009 (0.0015) C4 * -0.0003 (0.0014) C5 * -0.0016 (0.0014) C6 * -0.0046 (0.0015) C7 * 0.0104 (0.0015) C8 * 0.0089 (0.0015) C9Rms deviation of fitted atoms = 0.0082
Refinement. Refinement of *F*^2^ against ALL reflections. The weighted *R*-factor *wR* and goodness of fit *S* are based on *F*^2^, conventional *R*-factors *R* are based on *F*, with *F* set to zero for negative *F*^2^. The threshold expression of *F*^2^ > σ(*F*^2^) is used only for calculating *R*-factors(gt) *etc*. and is not relevant to the choice of reflections for refinement. *R*-factors based on *F*^2^ are statistically about twice as large as those based on *F*, and *R*- factors based on ALL data will be even larger.

### Fractional atomic coordinates and isotropic or equivalent isotropic displacement parameters (Å^2^)

**Table:**

	*x*	*y*	*z*	*U*_iso_*/*U*_eq_	
Br	0.41731 (5)	0.68583 (2)	−0.065128 (12)	0.03136 (9)	
N	0.2730 (3)	0.49455 (15)	0.40535 (10)	0.0155 (3)	
H01	0.131 (5)	0.545 (3)	0.4306 (17)	0.025 (6)*	
O	0.2033 (3)	0.33201 (12)	0.51915 (8)	0.0187 (2)	
C1	0.3423 (4)	0.36850 (17)	0.44644 (11)	0.0159 (3)	
C2	0.4140 (4)	0.54534 (17)	0.32544 (11)	0.0155 (3)	
C3	0.6381 (4)	0.46744 (18)	0.28064 (11)	0.0168 (3)	
H3	0.7347	0.5018	0.2253	0.020*	
C4	0.7296 (4)	0.33229 (17)	0.31716 (11)	0.0162 (3)	
C5	0.9617 (4)	0.24635 (19)	0.27310 (12)	0.0194 (3)	
H5	1.0630	0.2778	0.2178	0.023*	
C6	1.0421 (4)	0.11699 (19)	0.30999 (12)	0.0216 (4)	
H6	1.1988	0.0603	0.2798	0.026*	
C7	0.8955 (4)	0.06803 (18)	0.39159 (12)	0.0216 (4)	
H7	0.9509	−0.0217	0.4160	0.026*	
C8	0.6701 (4)	0.15123 (18)	0.43607 (12)	0.0190 (3)	
H8	0.5721	0.1192	0.4918	0.023*	
C9	0.5853 (4)	0.28290 (17)	0.39932 (11)	0.0159 (3)	
C10	0.2940 (4)	0.68958 (18)	0.29851 (12)	0.0194 (3)	
H10A	0.3169	0.7560	0.3474	0.023*	
H10B	0.0811	0.6853	0.2937	0.023*	
C11	0.4378 (4)	0.74776 (17)	0.21028 (12)	0.0170 (3)	
C12	0.3752 (4)	0.69677 (18)	0.12761 (12)	0.0201 (3)	
H12	0.2438	0.6235	0.1261	0.024*	
C13	0.5061 (4)	0.75386 (19)	0.04767 (12)	0.0211 (3)	
C14	0.6969 (4)	0.8617 (2)	0.04693 (13)	0.0237 (4)	
H14	0.7836	0.9000	−0.0086	0.028*	
C15	0.7580 (4)	0.91238 (19)	0.12966 (14)	0.0247 (4)	
H15	0.8878	0.9864	0.1309	0.030*	
C16	0.6308 (4)	0.85559 (18)	0.21038 (12)	0.0195 (3)	
H16	0.6758	0.8906	0.2665	0.023*	

### Atomic displacement parameters (Å^2^)

**Table:**

	*U*^11^	*U*^22^	*U*^33^	*U*^12^	*U*^13^	*U*^23^
Br	0.04174 (14)	0.03719 (14)	0.01477 (12)	0.00054 (9)	−0.00234 (8)	−0.00284 (8)
N	0.0173 (7)	0.0153 (6)	0.0130 (6)	0.0004 (5)	0.0017 (5)	−0.0002 (5)
O	0.0218 (6)	0.0182 (6)	0.0152 (6)	−0.0004 (4)	0.0016 (5)	0.0024 (4)
C1	0.0177 (8)	0.0156 (7)	0.0148 (8)	−0.0021 (6)	−0.0031 (6)	−0.0007 (6)
C2	0.0184 (8)	0.0156 (7)	0.0130 (7)	−0.0029 (6)	−0.0020 (6)	−0.0008 (6)
C3	0.0186 (8)	0.0182 (8)	0.0134 (8)	−0.0027 (6)	−0.0002 (6)	0.0001 (6)
C4	0.0166 (7)	0.0173 (8)	0.0152 (8)	−0.0021 (6)	−0.0031 (6)	−0.0027 (6)
C5	0.0186 (8)	0.0226 (8)	0.0168 (8)	−0.0003 (6)	−0.0013 (6)	−0.0030 (6)
C6	0.0211 (8)	0.0228 (8)	0.0212 (9)	0.0041 (7)	−0.0050 (7)	−0.0066 (7)
C7	0.0270 (9)	0.0175 (8)	0.0211 (9)	0.0031 (7)	−0.0083 (7)	−0.0022 (6)
C8	0.0231 (8)	0.0175 (8)	0.0170 (8)	−0.0015 (6)	−0.0042 (6)	−0.0003 (6)
C9	0.0172 (8)	0.0158 (7)	0.0151 (8)	−0.0013 (6)	−0.0036 (6)	−0.0014 (6)
C10	0.0243 (8)	0.0172 (8)	0.0156 (8)	0.0019 (6)	0.0015 (7)	0.0007 (6)
C11	0.0193 (8)	0.0136 (7)	0.0166 (8)	0.0037 (6)	0.0013 (6)	0.0014 (6)
C12	0.0229 (8)	0.0175 (8)	0.0193 (8)	−0.0007 (6)	−0.0004 (7)	0.0001 (6)
C13	0.0250 (9)	0.0220 (8)	0.0151 (8)	0.0051 (7)	−0.0005 (7)	−0.0001 (6)
C14	0.0246 (9)	0.0243 (9)	0.0202 (9)	0.0016 (7)	0.0045 (7)	0.0073 (7)
C15	0.0250 (9)	0.0205 (8)	0.0280 (10)	−0.0043 (7)	0.0009 (7)	0.0044 (7)
C16	0.0213 (8)	0.0179 (8)	0.0189 (8)	0.0012 (6)	−0.0016 (6)	−0.0003 (6)

### Geometric parameters (Å, °)

**Table:**

Br—C13	1.8997 (18)	C12—C13	1.384 (2)
N—C1	1.367 (2)	C13—C14	1.386 (3)
N—C2	1.379 (2)	C14—C15	1.390 (3)
O—C1	1.245 (2)	C15—C16	1.387 (3)
C1—C9	1.463 (2)	N—H01	0.85 (3)
C2—C3	1.352 (2)	C3—H3	0.9500
C2—C10	1.507 (2)	C5—H5	0.9500
C3—C4	1.438 (2)	C6—H6	0.9500
C4—C9	1.407 (2)	C7—H7	0.9500
C4—C5	1.413 (2)	C8—H8	0.9500
C5—C6	1.379 (3)	C10—H10A	0.9900
C6—C7	1.404 (3)	C10—H10B	0.9900
C7—C8	1.380 (3)	C12—H12	0.9500
C8—C9	1.403 (2)	C14—H14	0.9500
C10—C11	1.509 (2)	C15—H15	0.9500
C11—C16	1.394 (2)	C16—H16	0.9500
C11—C12	1.393 (2)		
			
C1—N—C2	125.36 (14)	C14—C15—C16	120.44 (17)
O—C1—N	120.74 (15)	C15—C16—C11	120.83 (17)
O—C1—C9	123.72 (15)	C1—N—H01	117.5 (16)
N—C1—C9	115.54 (15)	C2—N—H01	117.1 (16)
C3—C2—N	119.97 (15)	C2—C3—H3	120.2
C3—C2—C10	126.87 (15)	C4—C3—H3	120.2
N—C2—C10	113.15 (14)	C6—C5—H5	119.8
C2—C3—C4	119.66 (15)	C4—C5—H5	119.8
C9—C4—C5	118.38 (15)	C5—C6—H6	119.6
C9—C4—C3	119.57 (15)	C7—C6—H6	119.6
C5—C4—C3	122.06 (15)	C8—C7—H7	120.2
C6—C5—C4	120.36 (16)	C6—C7—H7	120.2
C5—C6—C7	120.90 (16)	C7—C8—H8	119.9
C8—C7—C6	119.53 (16)	C9—C8—H8	119.9
C7—C8—C9	120.25 (17)	C2—C10—H10A	108.5
C8—C9—C4	120.57 (16)	C11—C10—H10A	108.5
C8—C9—C1	119.53 (15)	C2—C10—H10B	108.5
C4—C9—C1	119.89 (15)	C11—C10—H10B	108.5
C2—C10—C11	114.88 (14)	H10A—C10—H10B	107.5
C16—C11—C12	119.00 (16)	C13—C12—H12	120.3
C16—C11—C10	120.36 (16)	C11—C12—H12	120.3
C12—C11—C10	120.63 (15)	C13—C14—H14	120.9
C13—C12—C11	119.41 (16)	C15—C14—H14	120.9
C12—C13—C14	122.10 (17)	C16—C15—H15	119.8
C12—C13—Br	119.42 (14)	C14—C15—H15	119.8
C14—C13—Br	118.46 (13)	C15—C16—H16	119.6
C13—C14—C15	118.22 (16)	C11—C16—H16	119.6
			
C2—N—C1—O	179.43 (15)	O—C1—C9—C8	0.6 (3)
C2—N—C1—C9	−0.6 (2)	N—C1—C9—C8	−179.41 (15)
C1—N—C2—C3	1.0 (3)	O—C1—C9—C4	179.80 (15)
C1—N—C2—C10	−178.22 (15)	N—C1—C9—C4	−0.2 (2)
N—C2—C3—C4	−0.5 (2)	C3—C2—C10—C11	2.2 (3)
C10—C2—C3—C4	178.55 (16)	N—C2—C10—C11	−178.69 (14)
C2—C3—C4—C9	−0.2 (2)	C2—C10—C11—C16	−106.28 (18)
C2—C3—C4—C5	179.80 (16)	C2—C10—C11—C12	75.1 (2)
C9—C4—C5—C6	0.4 (2)	C16—C11—C12—C13	0.1 (2)
C3—C4—C5—C6	−179.64 (16)	C10—C11—C12—C13	178.82 (15)
C4—C5—C6—C7	0.1 (3)	C11—C12—C13—C14	−0.6 (3)
C5—C6—C7—C8	−0.8 (3)	C11—C12—C13—Br	−179.29 (12)
C6—C7—C8—C9	0.9 (3)	C12—C13—C14—C15	0.4 (3)
C7—C8—C9—C4	−0.4 (3)	Br—C13—C14—C15	179.16 (13)
C7—C8—C9—C1	178.80 (15)	C13—C14—C15—C16	0.1 (3)
C5—C4—C9—C8	−0.2 (2)	C14—C15—C16—C11	−0.6 (3)
C3—C4—C9—C8	179.78 (15)	C12—C11—C16—C15	0.4 (3)
C5—C4—C9—C1	−179.46 (15)	C10—C11—C16—C15	−178.27 (16)
C3—C4—C9—C1	0.6 (2)		

### Hydrogen-bond geometry (Å, °)

**Table:**

*D*—H···*A*	*D*—H	H···*A*	*D*···*A*	*D*—H···*A*
N—H01···O^i^	0.85 (3)	1.96 (3)	2.8036 (19)	176 (2)

Symmetry codes: (i) −*x*, −*y*+1, −*z*+1.
